# A 3D-Printed Millifluidic Platform Enabling Bacterial Preconcentration and DNA Purification for Molecular Detection of Pathogens in Blood

**DOI:** 10.3390/mi9090472

**Published:** 2018-09-17

**Authors:** Yonghee Kim, Jinyeop Lee, Sungsu Park

**Affiliations:** School of Mechanical Engineering, Sungkyunkwan University, Suwon 16419, Korea; hanskim723@gmail.com (Y.K.); softmemsljy@naver.com (J.L.)

**Keywords:** immunomagnetic separation (IMS), bacterial pathogen, 3D printing, preconcentration, DNA purification, molecular diagnostics

## Abstract

Molecular detection of pathogens in clinical samples often requires pretreatment techniques, including immunomagnetic separation and magnetic silica-bead-based DNA purification to obtain the purified DNA of pathogens. These two techniques usually rely on handling small tubes containing a few millilitres of the sample and manual operation, implying that an automated system encompassing both techniques is needed for larger quantities of the samples. Here, we report a three-dimensional (3D)-printed millifluidic platform that enables bacterial preconcentration and genomic DNA (gDNA) purification for improving the molecular detection of target pathogens in blood samples. The device consists of two millichannels and one chamber, which can be used to preconcentrate pathogens bound to antibody-conjugated magnetic nanoparticles (Ab-MNPs) and subsequently extract gDNA using magnetic silica beads (MSBs) in a sequential manner. The platform was able to preconcentrate very low concentrations (1–1000 colony forming units (CFU)) of *Escherichia coli* O157:H7 and extract their genomic DNA in 10 mL of buffer and 10% blood within 30 min. The performance of the platform was verified by detecting as low as 1 CFU of *E. coli* O157:H7 in 10% blood using either polymerase chain reaction (PCR) with post gel electrophoresis or quantitative PCR. The results suggest that the 3D-printed millifluidic platform is highly useful for lowering the limitations on molecular detection in blood by preconcentrating the target pathogen and isolating its DNA in a large volume of the sample.

## 1. Introduction

It is important to accurately detect pathogens in clinical samples at very low concentrations [1,2]. Methods for detecting pathogens in clinical samples, such as blood and saliva, include bacterial culture and polymerase chain reaction (PCR) [3]. However, when detecting pathogens in clinical samples, there are limitations because of the presence of substances that inhibit PCR [3,4]. Thus, such methods of detection still require sample pretreatment to isolate the target microorganisms and purify their nucleic acids [3,4,5]. Among these pretreatment techniques, immunomagnetic separation (IMS) [5,6] and magnetic silica bead (MSB)-based DNA purification [7,8,9] are the most popular. However, these two technologies usually rely on handling small tubes containing several millilitres of the sample and manual operation; thus, there is an urgent need for automated systems that can process large volumes of the samples simultaneously because higher concentrations of purified DNA can be obtained by preconcentrating the pathogens and purifying DNA from larger volumes of samples.

In the past few decades, microfluidic devices (μFDs) have been developed as platforms that can detect pathogens [10,11,12]. In particular, μFDs offer several advantages for detection when integrating IMS. For example, μFDs have a large surface area, thus allowing antibody-conjugated magnetic nanoparticles (Ab-MNPs) and targeted bacterial cells to quickly bind to each other [13]. In addition, the magnetic interaction between the Ab-MNPs and permanent magnets is very strong in the thin microchannels of the μFDs, and the bacteria–Ab-MNP complexes can be trapped easily and quickly [14]. Recently, efforts have been made to extract and isolate target DNA using either MSBs or IMS in μFDs [7,8,9,15,16]. However, conventional μFDs [13,14,15,16] are typically not suitable for processing samples larger than 1 mL due to the small dimensions (~1 mm) of their microchannels. In addition, their fabrication requires multiple layers and several bonding steps, making them difficult to mass-produce. Most recently, we have demonstrated that a three-dimensional (3D)-printed µFD (3DpμFD) is an excellent platform for detecting pathogens because of its high speed, integration, and automation [17]. Compared to photolithography and soft lithography, 3D printing has many advantages when printing μFDs because this technique easily enables the printing of high aspect ratio structures and does not require complex binding steps to form a monolithic structure. In recent years, considerable efforts have been devoted to the development of 3D printing microfluidic platforms for separation and detection [17,18,19]. However, to the best of our knowledge, there have been no reports showing the integration of both IMS and DNA purification functions into a single device.

In the present study, we report a 3D-printed millifluidic device (3DpmFD) that can perform IMS and DNA purification of the target pathogen in 10 mL or higher volumes of samples. The performance of the 3DpmFD was tested with *Escherichia coli* O157:H7 and *Staphylococcus aureus* in a buffer and spiked blood samples. The performance of the device was verified by standard methods, such as colony counting, PCR, and quantitative PCR (qPCR).

## 2. Materials and Methods

### 2.1. 3D Printing of the Millifluidic Device

The millifluidic device was 3D-printed using a digital light processing (DLP) 3D printer (IM-96) (Carima Co., Seoul, Korea). This 3D-printing technique was based on photopolymerisation by emitting visible light at 405 nm onto a photocurable resin. The 3D sketch of the device was designed using the Student edition of Inventor^®^ Professional (Autodesk Inc., Seoul, Korea). This 3D sketch was cut into 100-µm-thick layers in the z-axis direction using the Carima Slicer software and was separated into 115 layers. Each layer was irradiated for 1.5 s. After the 3D-printing, the printed structure was washed with 70% ethanol for 5 min to eliminate any uncured resin. Then, the structure was solidified for 10 min using visible light to improve its mechanical strength. The entire process, including the solidification step, takes 30 min and does not require any additional assembly steps.

**Figure 1:**
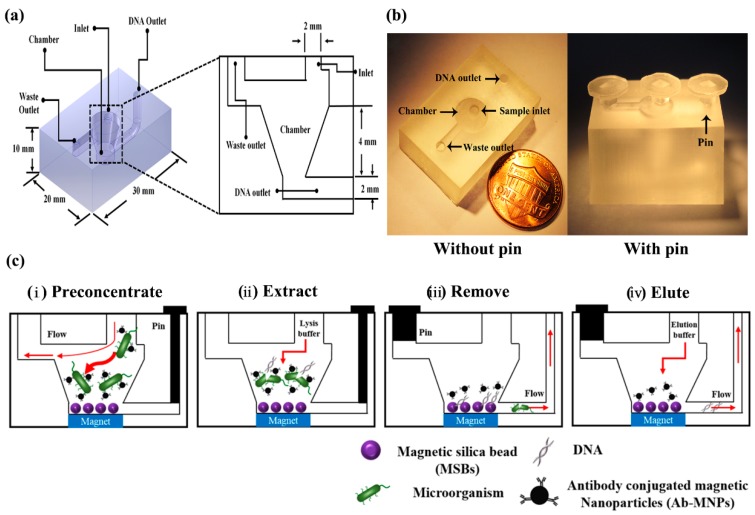
Bacterial preconcentration and genomic DNA (gDNA) purification on the three-dimensional (3D)-printed microfluidic device (µFD) (3DpmFD). (**a**) Design of the 3DpmFD. (**b**) A scale image of the 3DpmFD. (**c**) Schematic of the operational processes: (**i**) preconcentrating bacteria–Ab-MNP complexes; (**ii**) extracting gDNA from the complexes with lysis-binding buffer; (**iii**) removing buffer and bacterial debris by withdrawing flow using a pump; and (**iv**) eluting gDNA from MSBs with elution buffer.

Figure 1a,b show that the 3DpmFD (width × length × height dimensions of 20 × 30 × 10 mm^3^) consists of a cylindrical chamber (diameter: 5 mm, height: 2 mm) connected to two microchannels, a sample inlet (diameter: 2 mm), and DNA and waste outlets (diameter: 2 mm).

### 2.2. Bacterial Culture

The bacterial strains used in this study were *E. coli* O157:H7 (ATCC 43894) (American Type Culture Collection, Bethesda, MD, USA) and *S. aureus* (ATCC 29213) (ATCC, The strains were grown overnight in Luria broth (LB)) (Becton, Dickinson and Company, Franklin Lakes, NJ, USA) at 200 rpm and 37 °C. The culture was then diluted 100-fold with fresh LB and incubated again at 200 rpm and 37 °C until the optical density of the sample at 600 nm (OD_600_) was 1. Before preconcentration, the samples were serially diluted 10 times with phosphate-buffered saline (PBS) (pH 7.4).

### 2.3. Synthesis of Ab-MNPs

Amine-modified superparamagnetic nanoparticles (MNPs) of 50 nm diameter were purchased from Chemicell Co. (Berlin, Germany). The MNPs were sonicated for about 40 s to prevent aggregation. A solution of the MNPs (1 mg/mL) in PBS was then made to react with glutaraldehyde (2.5% *v*/*v*) in PBS at room temperature (RT) for 1 h using a rotary incubator and then washed with borate buffer (10 mM, pH 7.0) [20]. Aldehyde-functionalised MNPs were then mixed with 50 μg/mL of affinity purified anti-*E. coli* O157:H7 antibody (SeraCare Life Sciences Inc., Milford, MA, USA) or affinity purified anti-*S. aureus* antibody (SeraCare Life Sciences Inc.) in borate buffer and incubated at RT overnight. Next, Ab-MNPs were washed with 500 µL of borate buffer and then mixed with 1% bovine serum albumin (BSA) (Thermo Fisher Scientific, Waltham, MA, USA) in PBS to block unreacted aldehyde groups on the Ab-MNPs at RT for 1 h. To remove the unbound BSA, the Ab-MNPs were washed with 500 µL of borate buffer again. Then, the Ab-MNPs were treated with 20 mg/mL of sodium cyanoborohydride (Sigma-Aldrich, St. Louis, MO, USA) in borate buffer. Finally, the Ab-MNPs were washed with Tris-HCl buffer (pH 8.0) and stored in PBS at 4 °C until their use.

### 2.4. Effect of Flow Rates on Bacteria Capturing Efficiency in the 3DpmFD

Ten millilitres (10 mL) of PBS containing *E. coli* O157:H7 at 10^4^ colony-forming units (CFU)/mL were mixed with 200 µL of MNPs (10^13^ particles/mL, final concentration) conjugated with affinity-purified *E. coli* O157 antibodies and the mixture was incubated at 37 °C and 200 rpm for 20 min in a beaker. Then, the mixture was injected into the 3DpmFD through the sample inlet using a syringe pump (Harvard Apparatus, Boston, MA, USA) at various flow rates (1–10 mL/min) while placing a permanent magnet (diameter: 15 mm, height: 1.5 mm, magnetic flux density: 1720 G) underneath the chamber of the 3DpmFD.

The number of preconcentrated bacterial cells was estimated by counting the difference between the number of uncaptured bacterial cells and the total number of bacterial cells using the standard colony counting method [21]. Bacteria capturing efficiency was calculated using the following equation [17]:

Capturing efficiency (%) = (Nt − Ne)/Nt × 100%(1)

where Nt is the number of bacterial cells in the sample and Ne is the number of uncaptured bacterial cells in the sample.

### 2.5. Effect of MSB and Bacterial Concentration on DNA Purification

The 3DpmFD packed with MSBs (Chemicell Co., Berlin, Germany) was prepared by introducing 1 mL of PBS containing different concentrations (10^9^ to 5 × 10^10^ particles/mL) of MSBs at 2 mL/min through the sample inlet while placing a permanent magnet under the chamber and closing the DNA outlet with a plastic pin. Different volumes (1–100 mL) of PBS containing *E. coli* O157:H7 or *S. aureus* at various concentrations (1–10^5^ CFU/mL, final concentration) were first mixed with MNPs conjugated with antibodies specific to the pathogens and the mixture was incubated at 37 °C and 200 rpm for 20 min in a beaker. Then, the mixture was injected into the 3DpmFD packed with MSBs through the sample inlet using a syringe pump at 2 mL/min as shown in Figure 1c-i.

To lyse preconcentrated bacterial cells, 100 μL of Lysis and Binding buffer (Chemicell Co.) were loaded into the chamber as shown in Figure 1c-ii. The 3DpmFD was agitated by the automated mini-vibration system (DVM-N20 vibration motor, D&J WITH Co., Ltd., Seoul, Korea) at RT for 5 min and the blocking pins were removed from the 3DpmFD before placing it on the magnet. The buffer was removed from the chamber through the waste channel, and then 500 µL of washing buffer I (Chemicell Co.) and 500 µL of 70% ethanol were injected into the 3DpmFD sequentially. For the elution step, 50 μL of RNase free water was loaded into the 3DpmFD without placement on the magnet. After all the inlets and outlets were blocked with the pins again, the device was agitated on a thermo-shaker at 1200 rpm and 65 °C for 15 min. Finally, the DNA released from the MSBs was removed from the device through the DNA outlet.

### 2.6. Effect of Dilution Factor of Blood on Efficiency of Sample Preparation in 3DpmFD

The use of blood was approved by the Institutional Review Board (IRB) of the university (SKKU) and its approval number was SKKU 2017-11-006. Whole blood was purchased from Innovative Research, Inc. (Novi, MI, USA) and this product was treated with 0.1% K2 ethylenediaminetetraacetic acid (EDTA) to prevent blood coagulation. The hematocrit (hct) value was about 39%, which was measured using Fisherbrand^TM^ Microhematocrit capillary tubes (Fisher Scientific Co LLC, Pittsburgh, PA, USA) and a MicroHematocrit Centrifuge (Thomas Scientific Inc., Swedesboro, NJ, USA). Then, 1 mL of whole blood containing 10^5^ CFU/mL of *E. coli* O157:H7 was diluted to 10, 25, and 50% with PBS and 200 µL of Ab-MNPs.

### 2.7. Bacterial Preconcentration and DNA Purification in Spiked Blood Samples

Then, 1 mL of whole blood was mixed with 8 mL of PBS, 1 mL of *E. coli* O157:H7 (10–10^4^ CFU/mL), and 200 µL of Ab-MNPs. The subsequent procedures were the same as those for the bacterial preconcentration and DNA purification steps.

### 2.8. Detection of Bacteria by PCR and qPCR Samples

Purified bacterial genomic DNA (gDNA) was amplified by PCR, and the amplification of target genes was verified by gel electrophoresis. The primers that were designed to amplify the 150-base pairs (bp) of *eae* gene coding intimin adherence protein in *E. coli* O157:H7 consist of a forward primer (GGCGGATTAGACTTCGGCTA) and a reverse primer (CGTTTTGGCACTATTTGCCC). The 207-bp of *nuc* gene coding the thermonuclease of *S. aureus* consists of a forward primer (ACACCTGAAACAAAGCATCC) and a reverse primer (TAGCCAAGCCTTGACGAACT). The conventional PCR was performed using a PCR reagent by MJ MINI™ thermocycler (Bio-RAD, Hercules, CA, USA). The PCR products were separated in a 1.5% TAE (Tris base, acetic acid, and EDTA) (50 ×) agarose gel at 100 V for 30 min.

The qPCR was performed using LightCycler^®^ Nano (Roche, Basel, Switzerland), and its cycle threshold (Ct) value was determined. The same primer sets as those used for the PCR were used here.

## 3. Results and Discussion

### 3.1. Effect of Flow Rate on Bacteria Capturing Efficiency in the 3DpmFD

**Figure 2:**
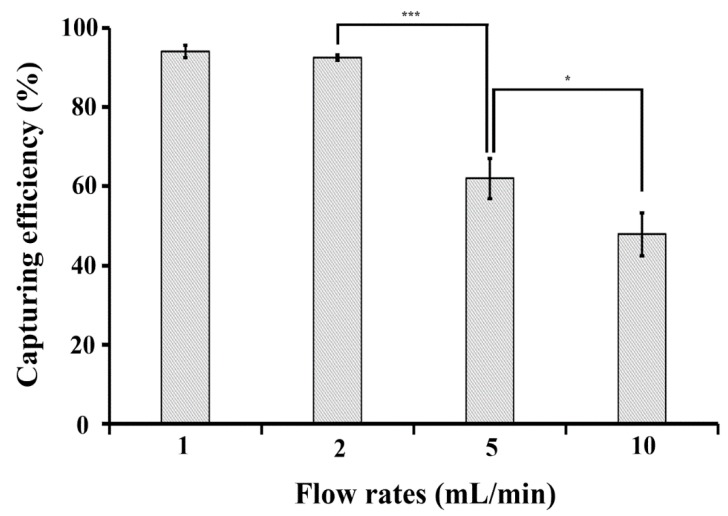
Effect of flow rate on bacteria capturing efficiency in 3DpmFD. Ten millilitres (10 mL) of PBS containing *E. coli* O157:H7 (10^4^ CFU/mL) and Ab-MNPs (10^13^ particles/mL) were injected into 3DpmFD at different flow rates (1–10 mL/min). *: *p* < 0.05, ***: *p* < 0.001. Student *t*-test. Sample number = 3.

The capturing efficiencies at flow rates of 1, 2, 5, and 10 mL/min were 94%, 92.5%, 62.1%, and 47.9%, respectively (Figure 2). The results show that it is difficult to capture bacteria–Ab-MNPs complexes at high flow rates, such as 5 mL/min and 10 mL/min, with the magnetic force of the permanent magnet located at the bottom of the device. Since there was no statistical difference in 1 mL/min and 2 mL/min, we selected 2 mL/min as a flow rate for the following experiments.

### 3.2. Optimisation of MSB Concentrations for DNA Purification using 3DpmFD

To find the optimal number for MSBs for DNA purification using the 3DpmFD, it was packed with different particle numbers (1 × 10^9^ to 5 × 10^10^) of MSBs before introducing 10 mL of PBS containing 10^3^ CFU/mL and Ab-MNPs containing 10^13^ particles/mL. All the bacteria capturing efficiencies were about 90%, suggesting that the number of MSBs does not affect the bacterial capturing efficiency on the 3DpmFD.

**Figure 3:**
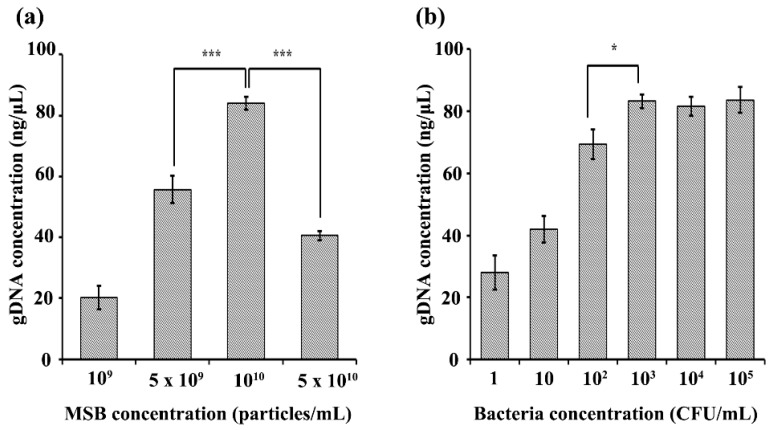
Effect of MSB and bacteria concentrations on DNA purification on the 3DpmFD: (**a**) gDNA concentration with different concentrations (10^9^–5 × 10^10^ particles/mL) of MSBs for 10^3^ CFU/mL of *E. coli* O157:H7; (**b**) gDNA concentration with different concentrations (1–10^5^ CFU/mL) of *E. coli* O157:H7 using 10^10^ particles/mL of MSBs *: *p* < 0.05, ***: *p* < 0.001. Student *t*-test. Sample number = 3.

Figure 3a shows that gDNA concentrations obtained using the 3DpmFDs increase as MSB concentrations in the range of 10^9^ to 10^10^ particles/mL increase. The maximum gDNA concentration was obtained using the 3DpmFD packed with 10^10^ particles/mL of MSBs. However, the gDNA concentrations at 5 × 10^10^ MSBs/mL were lower than those at 10^10^ MSBs/mL. This may be attributed to the fact that 50 μL of the elution buffer was not sufficient to fully wet the MSBs in the 3DpmFD. Thus, some DNA might not have been released from some of the MSBs, resulting in lower gDNA concentrations.

When the 3DpmFDs packed with 10^10^ MSBs/mL were injected along with 10 mL of PBS containing *E. coli* O157:H7 at different concentrations (1–10^5^ CFU/mL) at a rate of 2 mL/min, the gDNA concentration obtained using the 3DpmFDs increased as the bacterial concentrations increased in the range of 1 to 10^3^ CFL/mL, but did not increase any further at concentrations of 10^4^ and 10^5^ CFU/mL (Figure 3b), thus indicating that the surfaces of the MSBs were saturated with gDNA at such high concentrations. For such high concentrations, the sample solutions should be diluted to obtain accurate measurements.

### 3.3. Effect of Preconcentration and gDNA Purification Using 3DpmFD on Molecular Amplification of Genes in PBS

**Figure 4:**
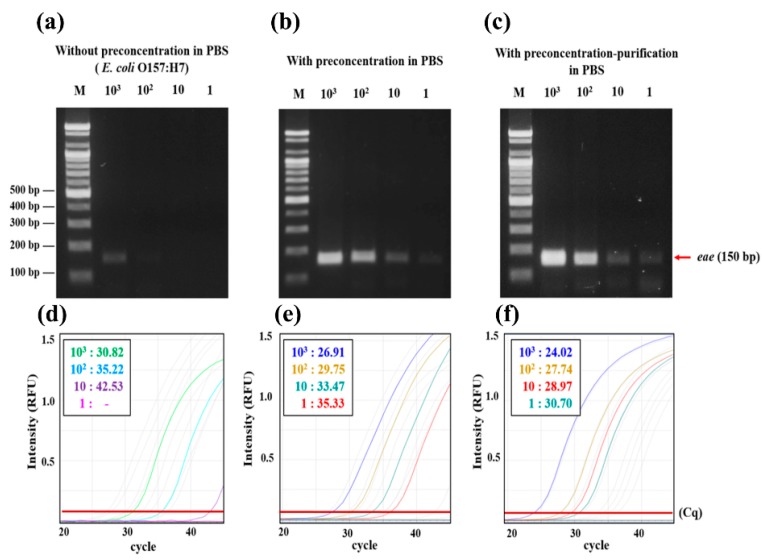
Confirmation of *E. coli* O157:H7 preconcentrating and DNA purifying efficiency using either PCR with gel electrophoresis and qPCR; (**a**) Gel electrophoresis of PCR products (*eae* gene) from 10 mL PBS containing *E. coli* O157:H7 at different concentrations (1–10^3^ CFU/mL) before preconcentration; (**b**) after preconcentration; (**c**) after bacterial preconcentration and DNA purification; (**d**–**f**) qPCR results of (**a**–**c**), respectively.

Once 10 mL of the Gram-negative pathogen *E. coli* O157:H7 at different concentrations (1–10^3^ CFU/mL) was preconcentrated and its gDNA was purified in a sequential manner using the 3DpmFD, the gDNA was amplified using either PCR or qPCR to verify the yield. The results were compared to those obtained from either untreated samples or samples prepared with only the preconcentration step using 3DpmFD. In the samples with the preconcentration–purification steps, a concentration as low as 1 CFU/mL was detectable using PCR with gel electrophoresis (Figure 4c), whereas in the untreated samples and samples with only the preconcentration step, concentrations as low as 10^3^ CFU/mL and 10 CFU/mL were detectable, respectively (Figure 4a,b). A similar trend was observed with qPCR. Considering that Ct values below 35 are reliable, a concentration as low as 1 CFU/mL was detectable in the samples with the preconcentration–purification steps using qPCR because its Ct value was 30.7 (Figure 4f). Concentrations as low as 10^3^ CFU/mL and 10 CFU/mL were respectively detectable in the untreated samples and samples with only the preconcentration step because their respective Ct values were 30.8 and 33.4 (Figure 4d,e). This remarkable improvement in the samples with the preconcentration–purification steps in detection using PCR and qPCR can be explained as follows. In the sample with only the preconcentration step, the Ab-MNPs can improve the detection by providing higher numbers of bacterial cells to the PCR and qPCR. However, Ab-MNPs and cell debris can inhibit DNA polymerase, so that the benefit offered by the bacterial preconcentration is decreased due to the modest interference from Ab-MNPs and cell debris in DNA amplification [22]. By including both preconcentration and purification steps in the 3DpmFD, only purified gDNA can be provided to the PCR and qPCR. This results in the enhancement in detection using PCR and qPCR.

**Figure 5:**
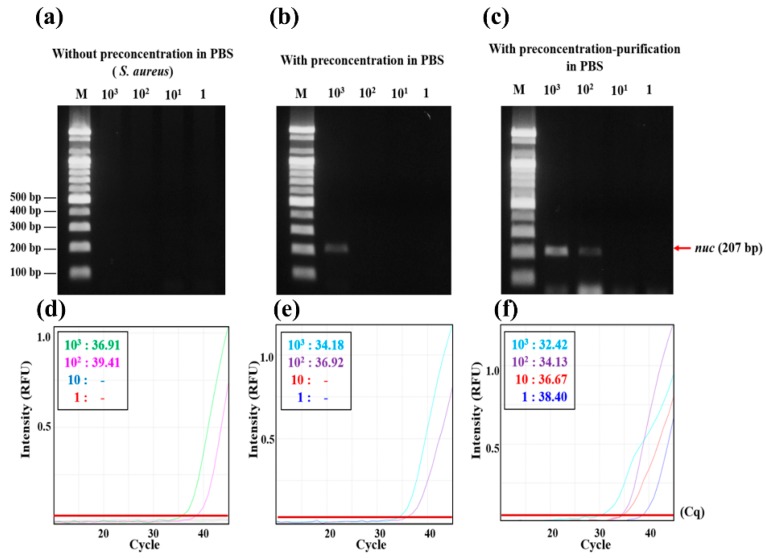
Confirmation of *Staphylococcus aureus* preconcentrating and DNA purifying efficiency using PCR with gel electrophoresis and qPCR; (**a**) Gel electrophoresis of PCR products (*nuc* gene) from 10 mL PBS containing *S. aureus* at different concentrations (1–10^3^ CFU/mL) before preconcentration; (**b**) after preconcentration; (**c**) after bacterial preconcentration and DNA purification; (**d**–**f**) qPCR results of (**a**–**c**), respectively.

The performance of the 3DpmFDs were further tested by preconcentrating the Gram-positive pathogen *S. aureus* at different concentrations (1–10^3^ CFU/mL) and purifying its gDNA using the 3DpmFD. Similar to the results (Figure 4) for *E. coli* O157:H7, significant improvements in the detection of *S. aureus* by both molecular diagnostics techniques, PCR post gel electrophoresis and qPCR, were observed in the samples with the preconcentration–purification steps. The lowest concentration for the detection in the samples with both preconcentration–purification steps using PCR post gel electrophoresis was 10^2^ CFU/mL (Figure 5c), whereas those for the samples with only the preconcentration step were 10^3^ CFU/mL (Figure 5b). However, *S. aureus* at all the concentrations (1–10^3^ CFU/mL) in the untreated samples was not detectable (Figure 5a). A similar trend was observed using qPCR. The cell walls of Gram-positive bacteria are thicker than those of the Gram-negative ones [7] and are hard to lyse by thermocycling of PCR. As a result, even at 10^3^ CFU/mL, the concentration of gDNA released from the lysed cells was not sufficient to be detected using PCR gel electrophoresis and qPCR. With the preconcentration step, bacterial numbers increased and their gDNA was detectable in the sample containing 10^3^ CFU/mL. Further improvement in the detection was possible when the preconcentration and purification steps were included in the 3DpmFD because the Lysis and Binding buffer in the DNA purification step induces cell lysis. Together with the results in samples containing *E. coli* O157:H7, these results suggest that gDNA purification is required as an addition in the 3DpmFD to improve the detection of bacterial pathogens using molecular diagnostic methods.

### 3.4. Dilution Effect on Preconcentration–Purification Efficiency in Blood Samples

We investigated the effect of dilution of blood on the preconcentration–purification efficiency by 3DpmFD. The bacteria capturing efficiency increased as the dilution factor increased. It was about 82% in the whole blood, while it was about 100% in 10% blood. These results suggest that the capturing efficiency was negatively affected by blood and blood dilution is required for the preconcentration step (Figure S1).

**Figure 6:**
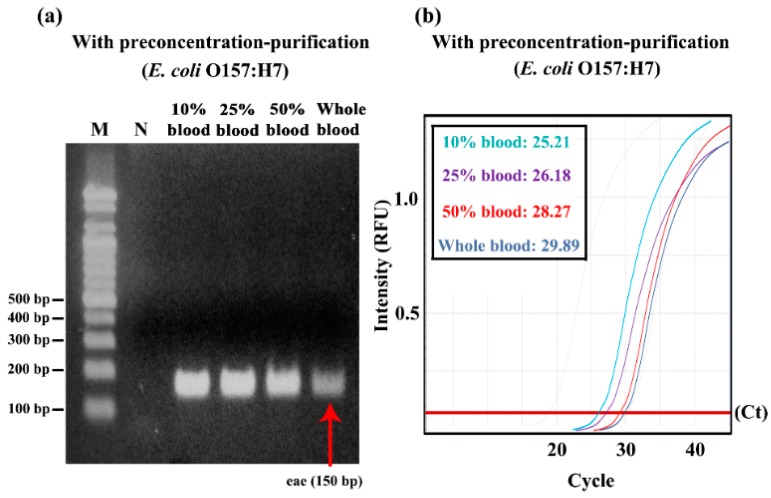
Confirmation of effect of dilution factor of blood on preconcentrating and gDNA purification efficiency with *E. coli* O157:H7 using PCR with gel electrophoresis and qPCR; (**a**) Gel electrophoresis of PCR products (*eae* gene) from different blood dilutions (10, 25, 50, and blood) containing 10^5^ CFU/mL of *E. coli* O157:H7 after bacterial preconcentration and DNA purification; (**b**) qPCR result.

Post gel electrophoresis with PCR (Figure 6a) shows that blood should be diluted at least 50% to detect 10^5^ CFU/ml of *E. coli* O157:H7 in the blood samples (Figure 6a). A similar trend was observed in the qPCR result (Figure 6b). There, 10^5^ CFU/ml of *E. coli* O157:H7 in all the diluted blood samples was detectable because their Ct values were lower than 35. However, Ct values increased as the ratio of blood in the diluted blood samples increased, suggesting that the ratio of blood should be lowered to 10%.

### 3.5. Spike Test in 10% Blood

**Figure 7:**
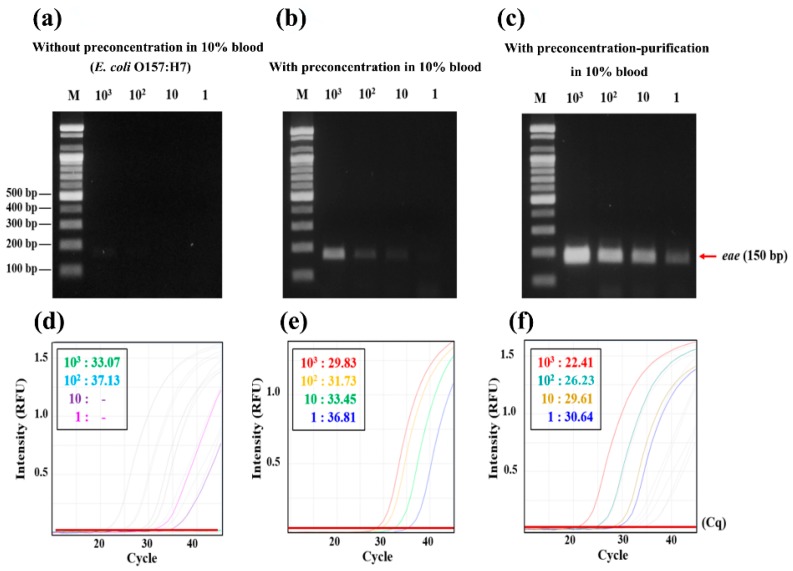
Confirmation of preconcentrating and DNA purification efficiency of *E. coli* O157:H7 in 10% blood using PCR with post gel electrophoresis after PCR (**a**–**c**) and qPCR (**d**–**f**). (**a**) Gel electrophoresis of PCR products (*eae* gene) from 10 mL of 10% blood containing *E. coli* O157:H7 at different concentrations (1–10^3^ CFU/mL) without preconcentration; (**b**) after preconcentrating; (**c**) after preconcentrating and DNA purification; (**d**–**f**) qPCR results of (**a**–**c**), respectively.

Blood has a lot of molecular diagnostic inhibitors, such as hematin and haemoglobin, and they prevent the action of Taq polymerase [23]. IgG in the blood also interferes with the activation of Taq polymerase as it interacts with single-strand DNA at high temperatures [24]. To minimize this inhibitory effect on molecular amplification, users have to use commercial kits and systems for purifying bacterial DNA from blood [25,26,27,28]. The QIAamp blood mini kit can purify DNA in 200 µL of blood without any dilution but cannot differentiate bacterial DNA from the DNA of blood cells. Microfluidic devices are not suitable for processing a large volume of samples, such as 10 mL, due to its slow flow rates in the range of 1–10 µL/min [27,29]. The hollow spinning disk system does not require the dilution of blood samples but only about 40% of bacteria can be isolated from the plasma by the system [30]. Figure 7 shows that the performance of the 3DpmFD for the sample preparation from 10 mL of 10% diluted blood with *E. coli* O157:H7 is excellent because *E. coli* O157:H7 can be detected for a concentration of up to 1 CFU/mL with the preconcentration and purification steps, which is 100 and 10 times lower than without sample treatment (Figure 7a,b). These results are also confirmed by qPCR through the difference between the Cq values of the samples (Figure 7d–f). The Cq value of the 1 CFU/mL is 30.6 and that of the 10^3^ CFU/mL without sample preparation is 33.8. These results suggest that the 3DpmFD can selectively preconcentrate the bacterial pathogen of interest and purify bacterial DNA from diluted blood samples.

For this reason, Figure 7a shows that the limit of detection is 10^3^ CFU/mL without sample preparation. Thus, it is very hard to detect pathogens in blood samples using PCR without any sample preparation steps. However, Figure 7c shows that the 3DpmFD can eliminate the molecular diagnostic inhibitors effectively. This is equivalent to a 50-fold improvement of sensitivity compared with the microfluidic device for the detection of *E. coli O157:H7* in 10% blood [31]. These results suggest that 3DpmFD is well-suited for improving the level of detection (LOD) in blood by including both bacterial preconcentration and DNA purification steps.

## 4. Conclusions

In this study, we report an improvement in detection for molecular diagnostics by successfully integrating both bacterial preconcentration and DNA purification in the 3DpmFD. The performance of the 3DpmFD shows that it is possible to preconcentrate very low concentrations of pathogens in 10 mL of 10% blood within 30 min, and 1 CFU/mL of *E. coli* O157:H7 can be detected by either PCR with post gel electrophoresis or qPCR. These results demonstrate that it is highly useful for improving molecular diagnostic methods in blood samples with the addition of the DNA purification step.

## Supplementary Materials

The following are available online at http://www.mdpi.com/2072-666X/9/9/472/s1, Figure S1: The effect of the dilution factor of blood on capturing efficiency with 10^3^ CFU/mL of *E. coli* O157:H7 *: *p* < 0.05, Student’s *t*-test. Sample number = 3.
