# Supplementary material for: A 3D-Printed Millifluidic Platform Enabling Bacterial Preconcentration and DNA Purification for Molecular Detection of Pathogens in Blood
Source: Micromachines (Basel). 2018 Sep 17;9(9):472. doi: 10.3390/mi9090472 (PMC6187281; doi:10.3390/mi9090472)
Supplement: Supplementary file 1 [file micromachines-09-00472-s001.pdf]

3D-printed millifluidic platform enabling bacterial preconcentration and DNA purification for molecular detection of pathogens in blood

Yonghee Kim[1+], Jinyeop Lee[1+] and Sungsu Park [1,*]

[1] School of Mechanical Engineering, Sungkyunkwan University, Suwon, 16419, Republic of Korea; nanopark@skku.edu

* Correspondence: nanopark@skku.edu; Tel.: +82-31-290-7431

Received: date; Accepted: date; Published: date

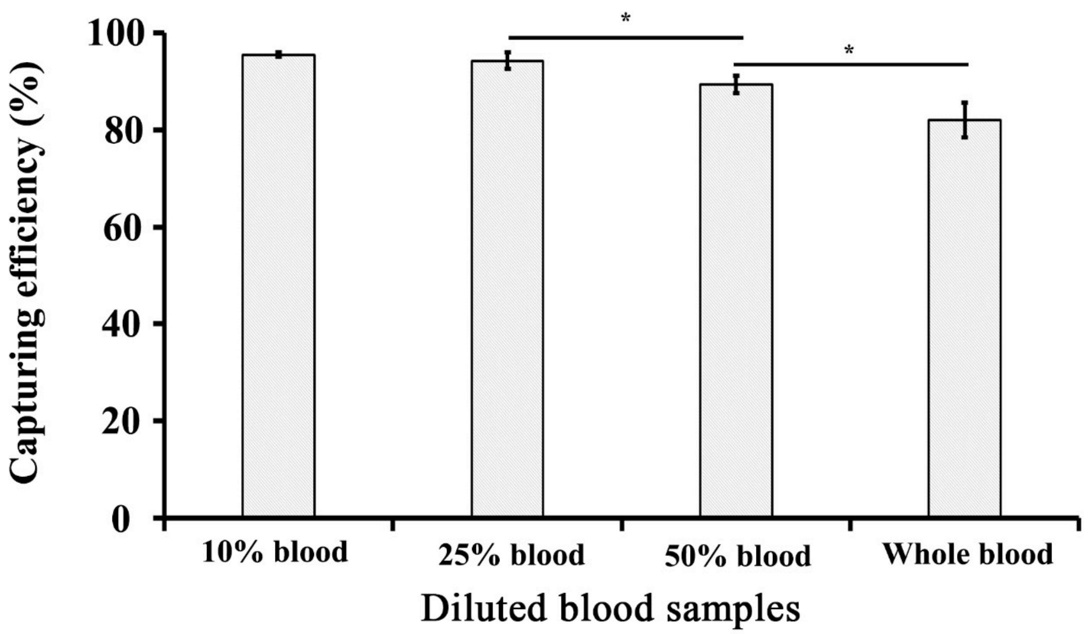

Figure S1. Effect of dilution of blood on bacterial capturing efficiency. 10 mL of either diluted blood (10%, 25%, 50%) or whole blood samples containing 10^5 CFU/mL of *E. coli* O157:H7 (final conc.) and Ab-MNPs (10^{13} particles/mL, final conc.) were injected into the 3DpmFD at a flow rate of 2 mL/min. *: $p < 0.05$, Student *t-test*. Sample number = 3.
